# Using a willingness to wait design to assess how readers value text

**DOI:** 10.1038/s41539-023-00160-3

**Published:** 2023-05-26

**Authors:** Amrita Bains, Carina Spaulding, Jessie Ricketts, Saloni Krishnan

**Affiliations:** 1 Department of Psychology, Royal Holloway, University of London, Egham Hill, Surrey, TW20 0EX UK; 2 The Reading Agency, 24 Bedford Row, London, WC1R 4EH UK

**Keywords:** Human behaviour, Reading

## Abstract

What affects moment-to-moment motivation to read? Existing reading motivation questionnaires are trait-based and not well suited to capturing the dynamic, situational influences of text or social context. Drawing on the decision science literature, we have created a paradigm to measure situational enjoyment during reading. Using this paradigm, we find reading enjoyment is associated with further decision-making about the text and with reading comprehension.

Reading plays a crucial role in determining academic success and life outcomes in literate societies^1,2^. Yet, reading is effortful, and people must make a choice to pursue reading. Being motivated to read is positively linked to vocabulary size^1^, decoding ability^2^, and comprehension^3^. But what motivates a reader to read? There are substantial within-individual fluctuations in reading motivation^4^. These are likely to be driven by transitory or situational factors, such as the text, social factors, and the purpose of reading. Traditional self-report questionnaires of reading motivation are not designed to identify these factors^5^, limiting insights for theory and educational practice. Self-report questionnaires also rely heavily on people’s memory of events, are prone to biases, and measure motivation only at the trait level. Here, drawing on work from the decision sciences, we develop and test a new measure to capture dynamic changes in reading enjoyment and motivation. We aim to capture the dynamic fluctuations in *enjoyment* related to the text presented.

The educational literature typically focuses on the difference between extrinsic and intrinsic motivation^3,6,7^. However, motivation can have multiple components^8,9^. In seminal neuroscientific work, Berridge and colleagues established that motivation involves at least two separable components: “liking”, or the experience of enjoyment or reward, and “wanting”, the desire to obtain a reward^8,10,11^. This framework highlights that people do not always pursue rewards. Indeed, rewards are discounted by the costs needed to obtain them. These costs can be monetary, physical, or even temporal. People’s decisions to take on such costs allow us to determine if a specific stimulus is considered desirable^12–15^. For instance, participants are willing to wait or pay for trivia they find interesting^15,16^. This neurobiological framework also aligns with educational motivation theories^9,17,18^, such as the situated expectancy-value theory^9^. According to the situated expectancy-value theory, subjective task value is influenced by task enjoyment, self-schema, later utility, and perceived costs, including effort, opportunity, or emotional costs of failure^9^. Yet, most reading motivation questionnaires only measure “liking” or enjoyment. Crucially, there is less focus on “wanting”. We aim to go beyond *enjoyment* to validate that participants *want* to engage with the text. We expect that measures of enjoyment will predict the likelihood of taking on costs, thereby establishing that enjoyment is indexing reward.

In the present study, we implemented a willingness to wait paradigm in two experiments. We measured participant enjoyment after reading synopses, allowing us to tap dynamic, situational changes in enjoyment. We then validated if rated enjoyment was linked to willingness to take on a cost (waiting for more information about the book). We also assessed how enjoyment predicted comprehension of the synopses. We hypothesised that higher enjoyment ratings would be associated with (1) a greater likelihood of waiting for more information about a book; and (2) higher comprehension scores.

In Experiment 1, participants (*N* = 40) encountered forty synopses of unfamiliar books. They rated their enjoyment of each synopsis. Next, they answered two questions that assessed their comprehension of the text. They were then presented with a choice to wait (for an unspecified period of time) if they wanted to find out more about the book. A wait time between 3 and 6 s was imposed if they chose “yes”, such that seeking information about the book was associated with a temporal cost (see Fig. 1 for a schematic). If they chose to wait, we showed participants the book cover, which provided details (book title, author, genre) that could be used to purchase the book later. After completing all 40 trials to assess dynamic changes in enjoyment, we obtained more typical trait-based measures of reading motivation. Participants completed the Adult Motivation for Reading Scale (AMRS)^19^, which is a self-report measure of intrinsic motivation. We asked about the time participants spent reading yesterday to measure reading engagement. Participants also completed a sentence verification task^20^, which allowed us to assess their reading ability (see *Methods* for more information). Experiment 2 was a direct replication of this experiment with a new sample of participants (*N* = 40).

**Fig. 1:**
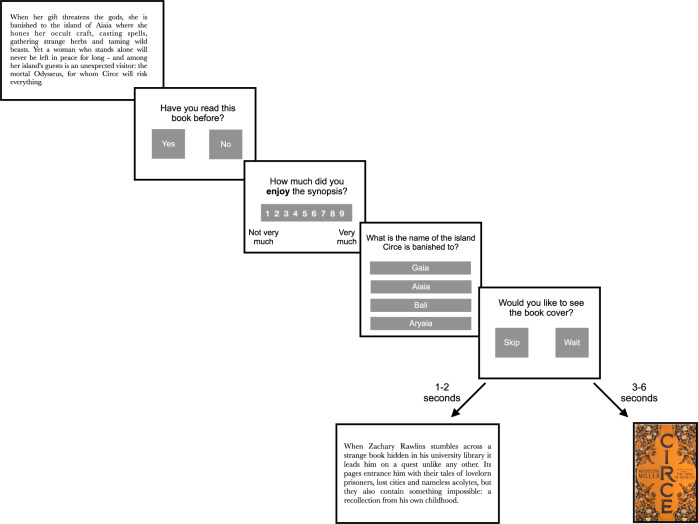
An illustration of a trial from the willingness to wait paradigm. Participants encountered a synopsis, stated whether they had read it previously, and then rated their enjoyment. Following this, they answered two multiple-choice comprehension questions. Finally, they were presented with the decision to wait to see the book cover. They could choose to either “wait” 3–6 seconds or “skip” initiating the next trial.

We used mixed effects logistic models to assess the contribution enjoyment made to willingness to wait and comprehension while accounting for variability across participants and items. In Experiment 1, as hypothesised, enjoyment of a synopsis predicted participants’ likelihood to wait to learn more information about the corresponding book, *β* = 0.94, SE = 0.11, *z* = 8.73, *p* < 0.001 (Fig. 2a). The model suggests that 1 standard deviation (SD) increase in enjoyment was associated with a participant being 2.57 times more likely to wait for more information, indicating the size of our effect. We replicated this finding in Experiment 2, *β* = 1.03, SE = 0.11, *z* = 9.40, *p* < 0.001 (Fig. 2b). Here, a 1 SD increase in enjoyment was associated with a participant being 2.80 times more likely to wait for a book cover.

**Fig. 2:**
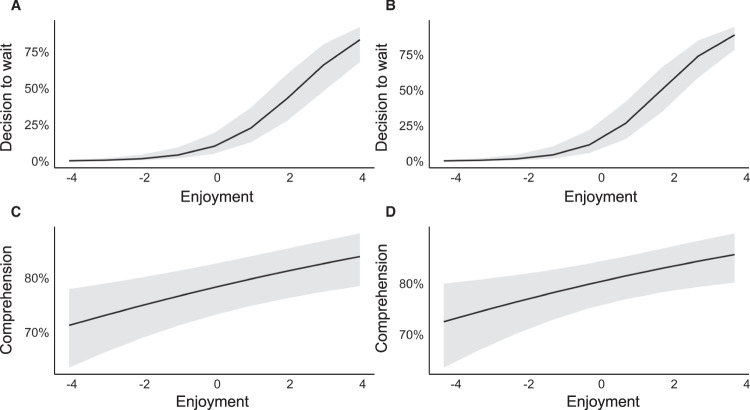
Enjoyment predicts the likelihood of waiting for more information about a book and the likelihood of answering a comprehension question accurately. Greater enjoyment is associated with a higher propensity to wait in Experiment 1 (**a**) and Experiment 2 (**b**). Enjoyment is positively associated with comprehension in Experiment 1 (**c**) and Experiment 2 (**d**). In all panels, the *x*-axes depict enjoyment ratings on a scale of −4 to 4 (due to the mean centering of the 1–9 scale). The *y*-axes in (**a**) and (**b**) illustrate the participant’s likelihood of waiting. The *y*-axes in (**c**) and (**d**) show the likelihood of answering a comprehension question accurately. In all panels, the solid black lines show the influence of enjoyment on the likelihood of waiting (**a**, **b**) and on the likelihood of providing an accurate answer (**c**, **d**). The shaded area around the solid line shows 95% confidence intervals.

Further, as hypothesised, enjoyment of a synopsis positively predicted comprehension in Experiment 1, *β* = 0.09, SE = 0.03, *z* = 3.25, *p* = 0.001 (Fig. 2c). A 1 SD increase in enjoyment led a participant to be 12 times more likely to correctly answer the comprehension questions. We replicated this finding in Experiment 2, *β* = 0.10, SE = 0.04, *z* = 2.71, *p* = 0.007 *(*Fig. 2d). A one standard deviation increase in enjoyment made a participant 1.11 times more likely to correctly answer the comprehension questions. In the model for Experiment 1, we controlled for reading ability, as this was significantly associated with comprehension, *r* = 0.36, *p* = 0.028. However, in Experiment 2, reading ability was not significantly associated with comprehension, *r* = −0.021, *p* = 0.9; therefore, based on our decision tree, reading ability was not included in the model. For further details on model construction, see *Methods*.

We then evaluated whether traditional measures of motivation (i.e., the AMRS^19^: Adult Motivation for Reading Scale) and reading engagement (time spent reading yesterday) correlated with our experimental measures—enjoyment, decision to wait, and comprehension. We pooled data across the two experiments to give us sufficient power to detect correlations. As expected, average enjoyment in our task was correlated with trait-based motivation as assessed by the AMRS, *r* = 0.27*, p* = 0.019. We did not find a correlation between average enjoyment in our task and reading engagement*, r* = 0.15, *p* = 0.21. Importantly, we found that decision to wait did not significantly correlate with reading engagement, *r* = 0.04, *p* = 0.76, or AMRS, *r* = −0.09, *p* = 0.43. Similarly, comprehension scores did not significantly correlate with reading engagement, *r* = −0.16, *p* = 0.17, or AMRS, *r* = −0.07, *p* = 0.54 (for Bayesian correlations, see Supplementary Material, Appendix 2).

Our study demonstrates the strong association between dynamic changes in enjoyment and the willingness to take on costs. Changes in enjoyment affect subjective value, as would be predicted by the situated expectancy-value theory^9^. Notably, we provided no extrinsic motivator to seek book covers; participants knew they would not be tested on their knowledge of book covers. The main cost participants incurred was their time. This cost was also a financial disincentive, as participants took longer to complete the experiment, and there was no additional compensation (we offered a fixed fee for participation). Yet, we found that participants were willing to wait when they enjoyed the synopsis. This has real-world implications. For example, when people enjoy text, they may be more likely to engage in “costly” behaviours such as paying for a book or choosing to spend time reading. This paradigm gives us a viable platform to ask these questions and establish whether the enjoyment is a fruitful target when designing reading interventions. This paradigm can be adapted in future research to assess other rewards, for example, a participant’s willingness to take on a cost to read the next paragraph of a book.

Somewhat surprisingly, we did not find a significant correlation between motivation scores reported on the AMRS^19^ and the likelihood of waiting. That is, individuals who self-identified as highly motivated readers were not more likely to wait to learn more information about a book than those with lower motivation scores. This was not due to a lack of variation in our sample or floor or ceiling effects (see Supplementary Material, Appendix 1). Further, AMRS^19^ scores were correlated with our enjoyment scores, showing that this measure did tap “liking”. This calls into question whether constructs tapped by traditional reading motivation questionnaires align with real-world decisions or whether these questionnaires are better conceptualised as participants’ perceptions of their reading motivation. We also did not find a significant correlation between reading engagement and waiting decisions, average enjoyment, or comprehension. We used a simple, concrete measure of time spent reading the previous day to measure reading engagement; for this reason, responses were positively skewed, which might explain the lack of a strong correlation with waiting decisions. Yet, reading engagement was correlated with AMRS^19^, suggesting that our measure did index reading engagement to a certain degree. However, other validated measures of reading engagement that use a wider timeframe may be more sensitive to variability in wait decisions^21,22^.

Enjoyment was associated with comprehension of a synopsis, even when controlling for individual reading ability. This suggests the link between enjoyment and comprehension is not driven by reading ability. This might initially seem contrary to findings from recent genetic studies, which suggest that reading ability is predictive of reading enjoyment rather than the other way around^23^. However, our work indicates that dynamic *states* of enjoyment—rather than the more stable traits typically tapped in genetic studies—are linked to comprehension. There is considerable research investigating how states of enjoyment turn into more stable traits over time, becoming a long-term interest^24,25^. Such long-term interests show important links to reading engagement^25^ and academic achievement^26^. However, we argue it is also important to understand *states* of enjoyment, as there are situations where a poor reader will enjoy reading. Indeed, in the neuroscience literature, the idea that states of high reward or motivation are linked to better learning is well established, with data showing enhanced coupling between reward and memory systems in states of high intrinsic and extrinsic reward^27–29^. This also has implications for education, as it suggests we could design targeted intervention strategies focusing on reading enjoyment to promote positive reading behaviours. Focusing on building states of high enjoyment could be an easier target during intervention than changing people’s self-perception of themselves as readers. We need future research to establish whether boosting enjoyment could improve comprehension and how such effects generalise to poor readers.

To summarise, using a new willingness to wait paradigm, we captured dynamic changes in enjoyment during reading. We demonstrated that these meaningfully predicted whether people would take on temporal costs during reading. We also found that higher levels of enjoyment were linked to improved comprehension of a synopsis. This paradigm now offers an elegant experimental means of exploring the factors that influence our enjoyment and motivation for reading.

## Methods

The Central Ethics Committee at Royal Holloway, University of London, reviewed and approved this study [ethical approval code: 2543-2021-02-05-17-21-PJJT001]. All participants provided written informed consent at the start of the experiment.

### Determination of sample size

A power analysis using the SimR^30^ package was used to determine the sample sizes for Experiment 1 and Experiment 2. To determine the power for Experiment 1, we used data from a pilot study (*n* = 23) which employed a similar, but not identical, design. This analysis indicated that willingness to take on a cost was a large effect, with an odds ratio of 14.06. That is, an increase of 1 standard deviation in enjoyment was associated with being 14 times more likely to see the cover of a book. For comprehension, the odds ratio was 1.36, which suggested that if enjoyment increased by 1 SD, the reader would be 1.36 times more likely to correctly answer the comprehension questions. We chose our sample size to have 90% power to detect an odds ratio of 1.36; this was established by using simr with the pilot data and estimating the variance–covariance matrix.

For Experiment 2, we used the data from Experiment 1 to determine the power of our hypotheses. For willingness to wait, the odds ratio was 6.57; that is, an increase of 1 standard deviation in enjoyment was associated with participants being 6.57 times more likely to wait and see the book cover. For comprehension, the odds ratio was 1.2; that is, if enjoyment increased by 1 standard deviation, participants were 1.2 times more likely to correctly answer the comprehension questions. Though the effect of enjoyment on comprehension appears to be small, in real-world settings, people encounter longer texts where the chance of making an error is greater. Therefore, we expect this effect to build up and have practical significance. For 90% power and an alpha of .05, simulations revealed we needed a sample of 5 for Hypothesis 1 and 40 for Hypothesis 2.

### Participants

We recruited participants using the Prolific platform, www.prolific.ac (see Supplementary materials for further details). We recruited 40 participants for Experiment 1 (*M*_age_ = 32.38 years, SD = 9.34, 29 females) and a new sample of 40 participants for Experiment 2 (*M*_age_ = 24.98 years, SD = 3.05, 20 females). Our inclusion criteria were adults aged between 18 and 50; however, in Experiment 1, one participant did not disclose their age and was later found to be 51. We excluded participants who reported any known neurodevelopmental conditions or any neurological disorders.

### Tasks

Participants completed the AMRS^19^, the reading engagement question, and the sentence verification task^20^, followed by the willingness to wait task. All tasks were completed online through the experimental platform Gorilla.sc^31^.

### Adult Motivation for Reading Scale

Trait-based motivation for reading was measured using the Adult Motivation for Reading Scale or AMRS^19^. This 21-item scale has good internal consistency (alpha = 0.85) and correlates to enjoyment of reading and time spent reading. It can be decomposed into four factors, Reading as Part of the Self, Reading Efficacy, Reading for Recognition and Reading to do well in other realms. To our knowledge, this was the only easily available and published questionnaire suitable for adults^5^.

### Reading engagement

We asked participants what they had read and how much time they spent reading one day prior to the experiment. They were given four options 0–30 min, 30 min to 1 h, 1–2 h or more than 2 h. In a pilot study, we established that completing this questionnaire 3 times did not yield substantially different information. During analysis, the four options above were coded as 1–4. These levels were then correlated with an individual’s likelihood to wait, enjoyment, and comprehension scores.

### Sentence Verification Task

We asked participants to complete a sentence verification task^20^, which provides an index of their reading ability (comprehension and fluency). The task consisted of 80 sentences. Each sentence stayed on the screen for a maximum of three seconds, during which time participants were asked to decide whether the sentence was either true or false. The statements were simple sentences based on real-world knowledge, for instance, “Grass is green”. Sentence length increased in later blocks, therefore, task difficulty also increased. For each correct response, the participant was given 1 point, with 80 points being the maximum score. Participants had 90 seconds in total to read and verify as many sentences as possible.

### Willingness to wait task

During the task, participants encountered forty synopses. Synopses were taken verbatim from a popular online book merchant. We avoided bestsellers or award-winning books while sampling a variety of genres across fiction and non-fiction books. We imposed a minimum word count of 60 and a maximum word count of 200. Flesch Kincaid reading ease scores for selected synopses were between 7.5 and 85.

Figure 1 shows an illustration of a trial. Participants were allowed a maximum of 1 min to read a synopsis. To judge familiarity, participants were asked whether they had read the book previously. They then rated how much they enjoyed reading the item on a scale from 1 (“hated it”) to 9 (“loved it”). To measure arousal, they were asked how tired they were on a scale of 1 (“very tired”) to 9 (“not tired at all”). Subsequently, they encountered two multiple-choice comprehension questions to answer, one literal and one which involved drawing inferences^32^. We piloted comprehension questions with a sample of 20 participants, ensuring no question was answered with >40% accuracy in isolation.

Participants were then given a choice to see the book cover of the synopses they read. They had two choices, either “skip” or “wait”. They were asked to select “skip” if they did not want to see the book cover, to move on to the next trial. Participants waited for 2s before moving on to the next trial. Participants were instructed to select “wait” if they wanted to find out more about the book (i.e., see the book cover). If they chose to wait, participants waited an additional 1–4s (on top of the 2s) before the book cover was revealed. The time delays for each cover to appear consequently varied between 3, 4, 5, or 6s. Time delays were counterbalanced for each synopsis across participants to ensure that delay did not specifically affect decisions. Our task included two trials to assess attention during the task. Participants read a short extract and answered one question about that extract. Participants were told the entire task was expected to take 1h, and they would be paid a fixed amount (£5.10).

### Statistical analyses

#### Exclusion criteria

We decided prior to data collection to exclude participants who failed either or both of our attention check trials. We also removed any items from the analysis where participants reported they had read the book before. Following data collection, we also excluded participants who chose to wait on all forty trials, as this suggested that they had not fully understood the task. For Experiment 2, we prospectively applied these criteria. Four participants were removed across both experiments; this did not affect the directionality of the results.

#### Analysis

All analyses were performed in R^33^. Mixed effect Logistic regression models were created using the lme4 package^34^ (see Brown et al., 2020 for further details about mixed effects models). Plots were created using the ggplot2^35^ and ggeffects package^36^.

We ran simple correlations between our experimental measures and traditional measures. We calculated metrics from our task for individual participants; the total number of wait decisions across 40 trials, the average enjoyment across 40 trials, and the number of questions correctly answered across 40 trials. We then correlated these with self-reported motivation from the AMRS, reading engagement measured by time spent reading yesterday, and reading ability measured by the sentence verification task. We included any of the traditional measures where correlations were a *p* < 0.2 into our models for likelihood to wait or comprehension.

*Hypothesis 1: Higher enjoyment ratings are associated with a greater likelihood to wait.*

We built a mixed effects logistic regression model with the decision to wait as the dependent variable and enjoyment as a fixed effect. To account for variation within individuals and items (i.e. synopses), we included participant and synopsis as random intercepts. We also included random slopes of enjoyment by synopsis and participant. Enjoyment ratings were mean-centred across participants prior to inclusion in the model. Consequently, the mean-centred scale had values between −4 and +4 and a mean of zero across participants. Across both studies, AMRS, reading ability, or reading engagement did not significantly correlate with the likelihood to wait, *p* > 0.2. We consequently did not include these as factors in our model. The model used was (1):

1 $${\rm{Wait}}\,{\rm{Choice}} \sim 1+{\rm{Enjoyment}}+(1+{\rm{Enjoyment}}| {\rm{Participant}})+(1+{\rm{Enjoyment}}| {\rm{Synopsis}})$$

*Hypothesis 2: Enjoyment ratings are positively associated with comprehension*.

Comprehension questions were scored either 0 or 1; therefore, we built a mixed effects logistic regression model with comprehension as the dependent variable and enjoyment as a fixed effect. To account for variation within individuals and items, we included random intercepts of participant and synopsis. Enjoyment ratings were mean-centred prior to model fitting. In both Experiment 1 and 2, comprehension was not significantly correlated with AMRS and reading engagement, *p* > 0.2; therefore, they were not included in either model. In Experiment 1, reading ability significantly correlated with comprehension (*p* < 0.05) and was included as a fixed effect in the model. As before, we included enjoyment as a random slope across participants and synopsis, but this model did not converge. Therefore, a simpler model was used (2):

2 $${\rm{Comprehension}} \sim 1+{\rm{Enjoyment}}+{\rm{ReadingAbility}}\,+(1|{\rm{Participant}})+(1|{\rm{Synopsis}})$$

In Experiment 2, reading ability and comprehension did not significantly correlate, *p* > 0.2; consequently, reading ability was not included as a fixed effect. We included enjoyment as a random slope across participants and synopsis, but this did not converge. A model with enjoyment as a random slope by participant was used (3):

3 $${\rm{Comprehension}} \sim 1+{\rm{Enjoyment}}+(1+{\rm{Enjoyment}}|{\rm{Participant}})+(1|{\rm{Synopsis}})$$

We also conducted exploratory analyses that assessed the influence of gender on wait decisions and comprehension (Supplementary Material, Appendix 3). Gender was not a significant predictor of either wait decisions or comprehension.

### Reporting summary

Further information on research design is available in the Nature Research Reporting Summary linked to this article.

## Supplementary information

Reporting Summary

Supplementary Material

## Data Availability

Data and scripts are openly available at the Open Science Framework: https://osf.io/ftexh/.
