# Supplementary material for: Using a willingness to wait design to assess how readers value text
Source: NPJ Sci Learn. 2023 May 26;8:17. doi: 10.1038/s41539-023-00160-3 (PMC10220033; doi:10.1038/s41539-023-00160-3)
Supplement: Supplementary file 1 — Reporting Summary [file 41539_2023_160_MOESM1_ESM.pdf]

nature portfolio

Corresponding author(s): Amrita Bains and Saloni Krishnan

Last updated by author(s): Mar 8, 2023

Reporting Summary

Nature Portfolio wishes to improve the reproducibility of the work that we publish. This form provides structure for consistency and transparency in reporting. For further information on Nature Portfolio policies, see our Editorial Policies and the Editorial Policy Checklist.

Statistics

For all statistical analyses, confirm that the following items are present in the figure legend, table legend, main text, or Methods section.

n/a	Confirmed	
☐	☒	The exact sample size (*n*) for each experimental group/condition, given as a discrete number and unit of measurement
☐	☒	A statement on whether measurements were taken from distinct samples or whether the same sample was measured repeatedly
☐	☒	The statistical test(s) used AND whether they are one- or two-sided<br>*Only common tests should be described solely by name; describe more complex techniques in the Methods section.*
☐	☒	A description of all covariates tested
☒	☐	A description of any assumptions or corrections, such as tests of normality and adjustment for multiple comparisons
☐	☒	A full description of the statistical parameters including central tendency (e.g. means) or other basic estimates (e.g. regression coefficient) AND variation (e.g. standard deviation) or associated estimates of uncertainty (e.g. confidence intervals)
☒	☐	For null hypothesis testing, the test statistic (e.g. *F*, *t*, *r*) with confidence intervals, effect sizes, degrees of freedom and *P* value noted<br>*Give P values as exact values whenever suitable.*
☒	☐	For Bayesian analysis, information on the choice of priors and Markov chain Monte Carlo settings
☒	☐	For hierarchical and complex designs, identification of the appropriate level for tests and full reporting of outcomes
☐	☒	Estimates of effect sizes (e.g. Cohen's *d*, Pearson's *r*), indicating how they were calculated

Our web collection on statistics for biologists contains articles on many of the points above.

Software and code

Policy information about availability of computer code

Data collection	We used gorilla.sc and Prolific.ac to collect data; no custom software was used
Data analysis	We used R version to analyse data; scripts are available on the OSF (https://osf.io/ftexh/)

For manuscripts utilizing custom algorithms or software that are central to the research but not yet described in published literature, software must be made available to editors and reviewers. We strongly encourage code deposition in a community repository (e.g. GitHub). See the Nature Portfolio guidelines for submitting code & software for further information.

Data

Policy information about availability of data

All manuscripts must include a data availability statement. This statement should provide the following information, where applicable:

- Accession codes, unique identifiers, or web links for publicly available datasets
- A description of any restrictions on data availability
- For clinical datasets or third party data, please ensure that the statement adheres to our policy

Data and scripts are openly available at the Open Science Framework: https://osf.io/ftexh/

Human research participants

Policy information about studies involving human research participants and Sex and Gender in Research.

Reporting on sex and gender	We collected information from participants about their gender, we report this information in the paper and this is also openly available in disaggregated format on OSF.<br><br>On request of the editor, we did conduct analyses to assess the influence of gender on our results. This is reported in Supplementary Materials (Appendix 3).
Population characteristics	See below (field specific reporting)
Recruitment	Participants were recruited via Prolific (standard sampling process for Experiment 1 and balanced sampling controlling for gender in Experiment 2).
Ethics oversight	We obtained approval from the Central Ethics committee at Royal Holloway, University of London.

Note that full information on the approval of the study protocol must also be provided in the manuscript.

Field-specific reporting

Please select the one below that is the best fit for your research. If you are not sure, read the appropriate sections before making your selection.

☐ Life sciences ☒ Behavioural & social sciences ☐ Ecological, evolutionary & environmental sciences

For a reference copy of the document with all sections, see nature.com/documents/nr-reporting-summary-flat.pdf

Behavioural & social sciences study design

All studies must disclose on these points even when the disclosure is negative.

Study description	This is a quantitative experimental study where we focus on reading motivation.
Research sample	Participants were recruited on Prolific.ac, we only recruited participants from majority English speaking countries (e.g., UK, USA). We recruited 18-50 year olds, further demographics are described in the methods section.
Sampling strategy	Details of our power analysis are reported in the methods; these determined our sample size. A convenience sample was recruited through Prolific.ac. For the second study, we balanced for gender.
Data collection	We collected data online through www.gorilla.sc
Timing	Data collection for Experiment 1 started on 13 May 2021 and ended on 20 May 2021. For Experiment 2, data collection started 26 April 2022 and finished 10 May 2022.
Data exclusions	All exclusions are clearly specified in the methods section.
Non-participation	In Experiment 1, 7 participants did not complete the experiment and were consequently not included. In Experiment 2, 8 participants did not complete the experiment and were consequently not included.
Randomization	N/A

Reporting for specific materials, systems and methods

We require information from authors about some types of materials, experimental systems and methods used in many studies. Here, indicate whether each material, system or method listed is relevant to your study. If you are not sure if a list item applies to your research, read the appropriate section before selecting a response.

Materials & experimental systems

n/a	Involved in the study
☒	☐ Antibodies
☒	☐ Eukaryotic cell lines
☒	☐ Palaeontology and archaeology
☒	☐ Animals and other organisms
☒	☐ Clinical data
☒	☐ Dual use research of concern

Methods

n/a	Involved in the study
☒	☐ ChIP-seq
☒	☐ Flow cytometry
☒	☐ MRI-based neuroimaging
