# Supplementary material for: Using a willingness to wait design to assess how readers value text
Source: NPJ Sci Learn. 2023 May 26;8:17. doi: 10.1038/s41539-023-00160-3 (PMC10220033; doi:10.1038/s41539-023-00160-3)
Supplement: Supplementary file 2 — Supplementary Material [file 41539_2023_160_MOESM2_ESM.pdf]

Supplementary Information

Appendix 1.

Supplementary table 1. Descriptive measures for Experiment 1.

	M	*S.D*	Range
Wait decisions	11.03	8.69	0-32
Enjoyment	5.08	1.99	1-9
Comprehension scores	59.76	7.83	33-69
Self-reported reading motivation (AMRS)	67.38	11.36	44-92
Sentence verification scores	56.68	10.21	36-74

Supplementary table 2. Descriptive measures for Experiment 2.

	M	*S.D*	Range
Wait decisions	11.31	9.46	0-38
Enjoyment	5.34	1.84	1-9
Comprehension scores	61.54	7.05	47-73
Self-reported reading motivation (AMRS)	65.33	9.69	46-88
Sentence verification scores	57.95	9.44	41-80

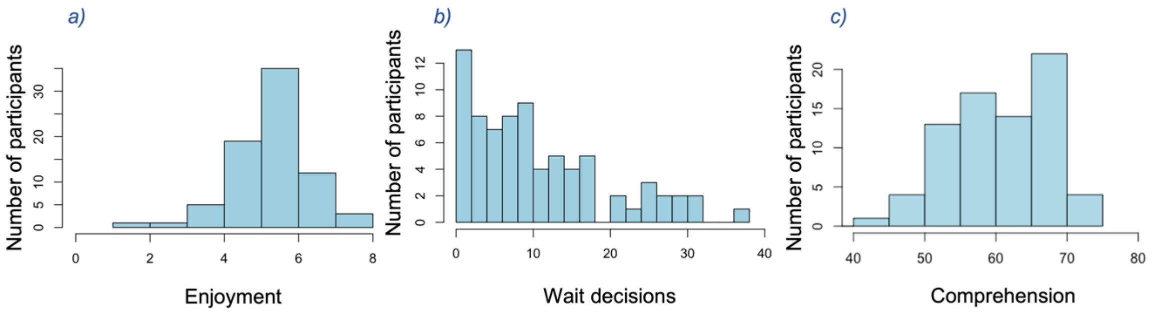

Supplementary figure 1. *Distributions of scores for A. enjoyment, B. number of decisions to wait, C. comprehension.*

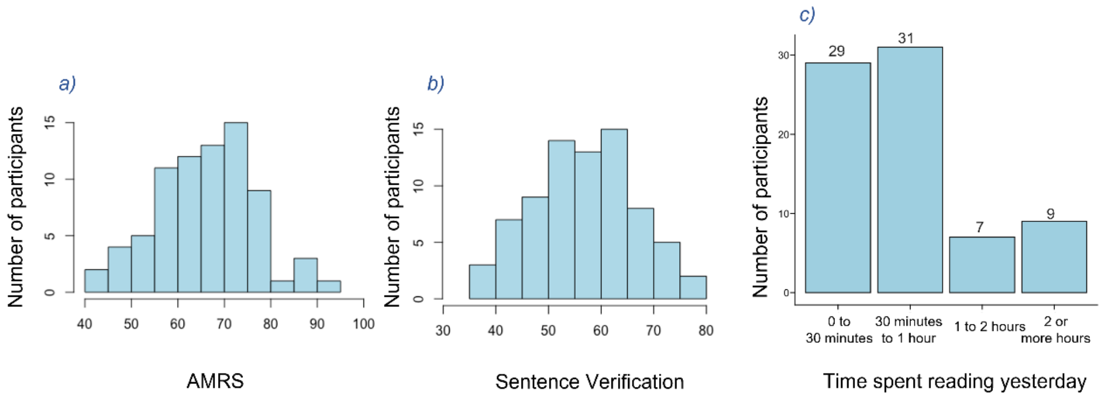

Supplementary figure 2. *Distributions for A. self-reported reading motivation from the AMRS, B. sentence verification scores and C. time spent reading.*

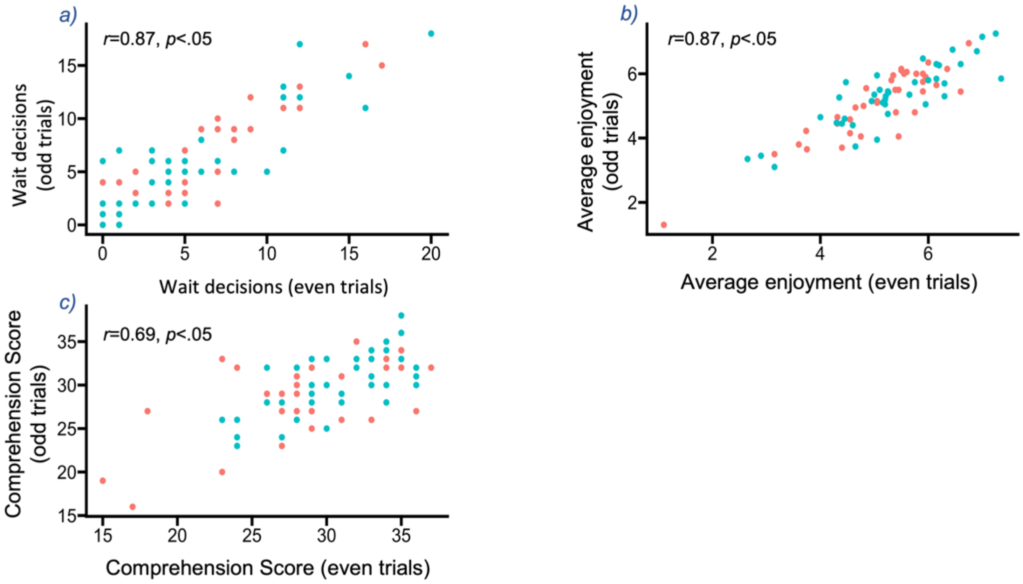

Supplementary figure 3. *Trials for each participant were split into two groups (trials labelled with an odd number or an even number) to assess internal reliability for our measures assessing A. the total wait decisions per participant, B. enjoyment ratings per participant and total accuracy for the comprehension questions correlating each score within each participant. Data points for Experiment 1 are shown in red and for Experiment 2 in blue.*

Appendix 2.

Supplementary table 3. Bayesian correlations show moderate evidence in favour of the null hypothesis for correlations between wait decisions and the AMRS, engagement and sentence verification scores. There is moderate evidence in favour of the null hypothesis for the correlation between comprehension and AMRS, and anecdotal evidence in favour of the null hypothesis for correlations between comprehension and engagement and sentence verification.

	Wait decisions	Comprehension
AMRS	0.31	0.31
Engagement	0.26	0.64
Sentence Verification	0.28	0.91

Appendix 3.

Influence of gender on likelihood of waiting and comprehension.

Likelihood of waiting

We investigated whether males and females differed in their likelihood to wait for more information about a book. Using data from Experiment 2, we reconstructed our model examining the effect of enjoyment on the likelihood to wait, including gender and the interaction between gender and enjoyment as fixed effects. the effect of as an interaction.

Decision to wait ~ Enjoyment + Gender + Gender*Enjoyment + (1 + Enjoyment | participant) + (1+ Enjoyment | synopsis)

As before, we observed a significant effect of enjoyment (p<.001). Gender did not emerge as a significant predictor of willingness to wait for more information about a book (p =.89) The interaction between enjoyment and gender was also not significant (p =.13).

Comprehension

We also investigated the influence of gender on comprehension. We included gender, and the interaction between gender and enjoyment, as fixed effects in our model examining the effect of enjoyment on comprehension.

Comprehension ~ Enjoyment + Gender + Gender*Enjoyment + (1 + Enjoyment | participant) + (1 + Enjoyment | synopsis).

As before, we observed a significant effect of enjoyment (p<.007). Gender did not emerge as a significant predictor of comprehension (p=.44). The interaction between enjoyment and gender was not significant (p=.2).
